# Supplementary material for: Ophiostomatales Associated with Mediterranean Pine Engraver, Orthotomicus erosus (Coleoptera, Curculionidae) in Dalmatia, Croatia
Source: J Fungi (Basel). 2022 Jul 28;8(8):788. doi: 10.3390/jof8080788 (PMC9410398; doi:10.3390/jof8080788)
Supplement: Supplementary file 1 [file jof-08-00788-s001.zip › jof-1794637-supplementary.pdf]

Table S1. Results of the blast analysis and GenBank accession numbers used in the study

**Species**	**No. of isolates**	**DNA barcode used for identification**	**GenBank Accession Number**	**Blast result (% Identity)**	**Accession Number of Reference Sequence**
Ophiostoma ips	30	ITS	ON697188	99-100%	MH324814.1
	5	β-tubulin (βT)	ON736861	98-100%	MT210369.1
	5	elongation factor 1-α (TEF 1-α)	ON736865	95-100%	MG702098.1
Sporothrix pseudoabietina	9	ITS	ON697189	99-100 %	KY568168
	9	β-tubulin (βT)	ON736862	95-99 %	MW066390.1
	9	elongation factor 1-α (TEF 1-α)	ON736866	99%	MW066432.1
Ophiostoma piceae	6	ITS	ON697190	99-100%	AF493240
	6	β-tubulin (βT)	ON736863	98-100%	AY789150.1
	6	elongation factor 1-α (TEF 1-α)	ON736867	97-100%	MH283420.1
Ophiostoma floccosum	3	ITS	ON697191	100%	JQ292830.1
	3	β-tubulin (βT)	ON736864	100%	MH124251.1
	3	elongation factor 1-α (TEF 1-α)	ON736868	100%	MH283416.1
Graphilbum rectangulosporium	5	ITS	ON697192	100%	KP691915.1
Sarocladium strictum	1	ITS	ON697193	100%	MF497404.1
